# Digital village construction, human capital and the development of the rural older adult care service industry

**DOI:** 10.3389/fpubh.2023.1190757

**Published:** 2023-07-20

**Authors:** Shengyou Liu, Shaopeng Zhu, Zhiping Hou, Changgui Li

**Affiliations:** ^1^Business School, Central South University, Changsha, China; ^2^Business School, Guilin University of Technology, Guilin, China; ^3^Management School, Guangzhou College of Technology and Business, Foshan, China

**Keywords:** digital village, human capital, rural older adult care service industry, threshold effect, coupling coordination evaluation

## Abstract

**Background:**

The advancement of digital villages in China is shaped by the degree of human capital within the rural labor force, which not only restricts the potential of digital village but also influences the impact of digital empowerment on the progression of the rural older adult care service industry.

**Materials and methods:**

Using panel data from 30 Chinese provinces between 2011 and 2020, we created benchmark and threshold regression models to investigate the influence of digital village construction on the development of the rural older adult care service industry and to delineate the threshold effects of human capital on it. We further scrutinized the correlation between the two systems, along with the factors that affect it, through a coupling coordination model.

**Results:**

Preliminary, the baseline regression outcomes show that the digital village construction is conducive to the progression of the rural older adult care service industry (*p* < 0.05). Moreover, we identified a significant nonlinear threshold relationship between the digital village, human capital, and the advancement of the rural older adult care service industry (HUM1_ST_, *p* < 0.05; HUM2_DT_, *p* < 0.01; HUM3_DT_, *p* < 0.01). These results indicate that the digital technology’s effect on the development of the rural older adult care service industry is limited by the rural human capital level. Lastly, we found that higher levels of human capital enhance the coupling of the digital village with the rural older adult care service industry (*p* < 0.01), with the influence of *per capita* education level being the most pronounced (*Coef*_HUM1_ > *Coef*_HUM2_ > *Coef*_HUM3_).

**Conclusion:**

The digital village substantially empowers the rural older adult care service industry, with human capital exhibiting a significant threshold effect on this empowerment. Furthermore, variances in the level of human capital have a considerable impact on the integration of the digital village and the rural older adult care service industry.

## 1. Introduction

As aging intensifies worldwide, the provision of older adult care poses a significant challenge for governments globally. Faced with the stark reality of “aging before affluence,” the Chinese government encounters even more severe trials. Research has indicated that rural regions present a higher degree of aging than urban areas (1), necessitating urgent solutions to China’s rural older adult care dilemma. Additionally, it highlights that in rural areas, the overall population is experiencing an aging trend, compounded by a decline in the younger population, which further contributes to the level of aging (2). Hence, rural older adult care has risen to prominence in Chinese government policies in recent years, becoming a primary concern for rural older adultly individuals and their offspring (3). Despite substantial resources and program support dedicated to rural older adult care, the policy impact still leaves considerable room for improvement. In addition, “off-target policies” have sporadically arisen. Certain scholars propose that this issue may be addressed by supporting increased provision and access to rural older adult care services (4). However, multiple factors have rendered the industry somewhat weak overall at present. Its market structure is imperfect, talent in older adult care products and services is scant, supply–demand balance remains elusive, effective supply is deficient, and both quality and efficiency are relatively low (5).

With the digital economy continues to advance rapidly, the enhancement of new infrastructure, represented by 5G base stations and fiber optic broadband networks, in rural China has paved the way for the synergistic integration of digital technology and rural older adult care services, marking a potential development trend (6). Digital older adult care services can curb transaction costs, boost the precision of supply–demand matching, and uplift the rural older adult care service industry’s performance by minimizing input factors. As such, the digital economy fuels endogenous growth (7, 8), that better meets the diversified (9). In response to the aging dilemma, the Chinese government has vigorously championed the establishment of information technology for older adult care services. This commitment is evident not only in the formation of the “National Expert Committee on Intelligent Aging” in 2013 but also in its consistent promotion of the intelligent aging industry in government reports spanning six consecutive years from 2014 to 2019. Moreover, in 2021, the Ministry of Industry and Information Technology, Ministry of Civil Affairs, and National Health and Welfare Commission jointly introduced the “Action Plan for the Development of an Intelligent Healthy Aging Industry (2021–2025),” setting a course for the evolution of China’s intelligent older adult care service industry and providing an unlocked an unprecedented developmental opportunity for its digital transformation subsequently. However, the digital enhancement is still in its pilot phase, with the full benefits of the digital economy not yet realized due to various constraints. Therefore, strategies for promoting the digital transformation of the rural older adult care service industry remain a pressing issue to be addressed.

Research findings indicate that digital villages infuse fresh dynamism into various economic and social activities, including but not limited to, economic development, inclusive digital finance, agricultural and rural digitalization, educational opportunities, and rural older adult care services (10). However, the efficacy of this digital empowerment is modulated by factors like the stage of economic development, extent of marketization, and level of human capital, among others. Notably, the degree of human capital plays a significant role in digital empowerment, thanks to the threshold effect associated with the use of digital technologies (11). For instance, a higher educational attainment by the head of the household is linked to a greater propensity to use digital payments for healthcare expenditures, leading to improved healthcare efficiency (12, 13). Moreover, enhancing the quality of human capital bolsters the digitalization impact on ameliorating income inequality (14, 15). A higher level of rural human capital translates into a more pronounced effect of digital financial inclusion on shaping the rural consumption structure (16).

Digital village construction, serving as a potent instrument for societal transformation, profoundly influences the enhancement of health services directed toward the rural older adults (15). This progression is increasingly conspicuous across rural regions of China. Illustrating it with Shapingba Village in Chongqing, China, the advancements in rural internet infrastructure have fostered an upsurge in the utilization and accessibility of healthcare facilities. A growing number of older adults in this region are now capable of conveniently accessing healthcare services and receiving bespoke health management counsel. This evolution markedly ameliorates the availability of medical resources for the aged populace while simultaneously improving their quality of life and overall health status. Consequently, the development of digital villages offers a critical opportunity to bridge the existing gap in digital care services for older adults in rural China (17). Smart older adult care, an outcome of the integration of digital technology with older adult care services (6), has successfully interconnected health data by fusing information technology and related products, thereby significantly enhancing the quality of its services (18). Nevertheless, several factors impede its development. These include low societal acceptance (19), an inadequate institutional environment (20), limited marketization (21), substandard infrastructure and service platforms (22), and a dearth of human resources (23). These factors can undermine the efficacy of digitally-powered older adult care in rural regions.

While current research delves extensively into the realms of digital villages and the rural older adult care service industry, the effect of the human capital threshold in the care service process has often been overlooked. The varying abilities of individuals to adopt and utilize digital technology can lead to disparate outcomes in the industry’s digital transformation, where these abilities largely hinge on their human capital (24, 25). Proceeding from this premise, we pose two critical research questions: To what extent does the variation in rural human capital impact the enabling influence of the digital village on the rural older adult care service industry? If such an effect exists, how does it manifest? In addressing these questions, this study aims to elucidate the pivotal role of human capital in digitally empowering the rural older adult care service industry, offering strategic suggestions for policymakers to enhance rural human capital levels and to optimize the use of digital technology. This paper distinguishes itself from existing research with two significant novelties. In terms of research content, it pioneers the exploration of the role of the human capital threshold effect in the digital empowerment of the rural older adult care service industry. In terms of research perspective, it provides a fresh perspective on the growth of the rural older adult care service industry by thoroughly examining the impact of human capital variances on its enabling effect.

This paper utilizes panel data from 2011 to 2020, encompassing rural areas across 30 provinces (municipalities) in China. We implement a panel threshold regression model to empirically examine the threshold effect of human capital on the digital empowerment of the rural older adult care service industry, with human capital serving as the threshold variable. This process enables us to determine the corresponding threshold values and establish specific quantitative relationships among the variables. Our findings elucidate the intricate interrelationship among digital villages, human capital, and the evolution of the rural older adult care service industry, thereby offering empirical evidence and theoretical insights for effectively implementing the rural revitalization strategy. The structure of the remainder of this article is as follows: Part II presents theoretical analysis and hypothesis development, Part III outlines the research design, Part IV delves into empirical analysis, Part V engages in further discussion, and finally, Part VI concludes the paper and elaborates on the implications.

## 2. Theoretical analysis and research hypothesis

### 2.1. Digital villages empower the rural older adult care service industry

According to the comprehensive index evaluation treatment of industrial development, the coordinated development of the rural older adult care service industry can be measured in four dimensions: infrastructure, service output, operating capital and basic security. The four dimensions, namely, physical support, efficiency, economic feasibility, and service quality, encapsulate the essence of the rural older adult care service industry’s development. They form the critical elements of the industry’s makeup. Consequently, an analysis of the digital rural enablement in the rural older adult care service industry, through these dimensions, provides a comprehensive understanding and evaluation of the industry’s current status and potential. Furthermore, this approach facilitates a precise exploration and representation of digital rural areas’ influence and impact on the industry’s development (26). Therefore, this paper analyzes the mechanism of the role of digital villages in empowering the rural older adult care service industry from these dimensions.

First, the construction of digital villages significantly enhances the infrastructure required for smart aging in rural areas. Current smart older adult care infrastructure typically comprises smart terminals, integrated service systems, and smart Wi-Fi (27). These facilities accommodate the diverse needs of older adults by synchronizing their information with a cloud-based platform (28). The availability of a regional network infrastructure is crucial for actualizing rural smart older adult care services. Digital villages can facilitate the widespread adoption of rural information network infrastructure, stimulate the transformation of traditional infrastructure, and broaden the application of digital technologies within the infrastructure for smart aging in rural areas (29). As the penetration rate of smartphones in rural areas increases, alongside greater broadband access and digital infrastructure coverage, the infrastructure constraints hampering rural smart aging start to diminish. This provides a solid foundation for incorporating numerous emerging technologies in the aging industry. Among them, network technologies have bolstered the hardware infrastructure for rural smart aging (30), satisfying older adults’ needs for services such as safety, independence, health, and assistance at an affordable cost (6). Additionally, the smart older adult care service platform is rooted in the smart older adult database system, which is developed using big data technology (31). This system effectively mitigates the information asymmetry issue in rural older adult care (32), providing software facility support for smart aging in rural areas.

Second, the construction of digital villages can enhance the input–output efficiency of rural older adult care services. The amalgamation of digital technology and older adult care services overcomes the technical and spatial constraints of traditional care services, eliminates information barriers between service providers and consumers, and eases the flow of information across various market nodes in older adult care services (33). This strategy not only enables real-time feedback on the distribution of care resources within the industry chain but also enhances the efficiency of resource allocation in the older adult care service industry (34). This process effectively accommodates the diversified needs of individuals in the industry and significantly alleviates the supply–demand mismatch in rural older adult care services (35). Specifically, the digital transformation of modern logistics and storage technologies in rural areas has made the distribution of older adult care end products increasingly efficient. Consequently, even consumers in remote areas can access care products that meet their needs without leaving their homes (36). Research indicates that when the internet penetration rate exceeds 15.9%, IT investment begins to substantially contribute to the improvement of the input–output ratio. This promotional effect of IT investment on technical efficiency improvement is further amplified when the internet penetration rate reaches 27.65% (37).

Again, the establishment of digital villages also fosters the accumulation of industrial capital within rural older adult care services. Digital technology provides a foundation for the automation and intelligence of business production, enabling machine substitution for manual labor, reducing labor input, and enhancing production efficiency (38). This transformation, in turn, elevates enterprise profitability (39). Furthermore, the substantial fixed costs and minimal marginal costs associated with digital technology can yield a “digital dividend” that showcases a significant scale effect (40). Moreover, a digital service platform for rural smart older adult care can significantly reduce information costs, transaction costs, and the costs associated with trial and error due to supply and demand mismatches, issues that are inherent in traditional older adult care services (41). Thus, compared to traditional older adult care services, digital technology decreases capital consumption in information transmission and logistics and distribution. This technology enhances return on assets in the rural older adult care service industry, fosters conditions conducive to the industry’s internal growth, and aids the expansion of operational capital within the rural older adult care sector (42).

Finally, digital village construction has enhanced the efficacy of safeguards in the rural older adult care service industry. Merging big data technology with rural older adult care service governance fosters conditions that encourage diverse stakeholders in the rural older adult care service industry to supervise its healthy development. Additionally, this fusion allows for the possibility of communal construction and shared governance within the rural older adult care service industry (43, 44). This multi-tiered governance system expands communication channels between government and public, refines the precision of rural pension policies and measures, upgrades the social security system, and assists in safeguarding the fundamental rights and interests of older adults (45). Therefore, digital technology’s intervention disrupts the uniformity of traditional rural older adult care service governance, prompting a shift from authoritative governance to technological governance. As a result, it fosters a holistic governance model predicated on the participation of various rural actors. The refinement of the rural older adult care governance system will make the development of the rural older adult care service industry more adaptable to the market and institutional environment conducive to the growth of advanced production factors. Based on the above analysis, the following hypothesis is proposed.

*H1*: The construction of digital villages significantly promotes the development and growth of the rural older adult care service industry.

### 2.2. Human capital heterogeneity in the digital village-empowered rural older adult care service industry

Insufficient human capital not only hinders economic development but also restricts the effectiveness of emerging technologies in revitalizing traditional industries (46). In the digital era, human capital emerges as a crucial factor shaping a regional “digital capability divide” and elucidating the impacts of digital empowerment (47–49). Although digital technology integration into real sectors could potentially stimulate knowledge spillovers, fostering the development of rural human capital (24, 50, 51), the effects of digital technology diffusion and application in rural areas remain limited. This limitation is largely due to the ongoing “hollowing out” of rural areas in China, in other words, the loss of younger populations due to depopulation or urbanization, which exacerbates the loss of rural human capital. Consequently, with the increasing penetration of digital technology into the “rural older adult” sector, human capital emerges as a key impediment to the growth of China’s rural smart older adult care service industry (52).

Human capital, defined by the accumulation of individual attributes such as knowledge, skills, cultural and technical proficiency, and health status, exhibits considerable variability across rural China, a variance attributable to a myriad of factors. Particularly in the extensive central and western regions, the local labor force’s low literacy and digital literacy levels, combined with their weak digital awareness and limited capabilities to utilize digital resources and technology, hinder their use of digital platforms for gathering and disseminating information on older adult care services. This constitutes a substantial barrier to the digital transformation of the rural older adult care service industry in these regions. On the contrary, the generally higher literacy levels of the urban populace in suburban areas of eastern coastal provinces and cities facilitate rapid learning and application of digital technology across various sectors. This active engagement promotes the sharing of digital technology and fosters knowledge spillover effects, expediting the digital transformation of the rural older adult care service industry (53). Consequently, while rural human capital level furnishes talent support for the rural older adult care service industry’s development, it simultaneously restricts the influence of rural digitalization. Notably, the impact of rural digitalization on the industry’s development exhibits heterogeneity, determined by varying rural human capital levels. Based on this analysis, the following hypothesis is proposed.

*H2*: The digital empowerment of the rural older adult care service industry has a human capital threshold effect. Higher levels of rural human capital tend to yield greater benefits from rural digitalization in promoting the development of this industry.

## 3. Study design

### 3.1. Model setting

#### 3.1.1. Baseline regression model

To test Hypothesis 1, the following benchmark model is constructed.


(1) $${\mathrm{IND}}_{\mathrm{it}}=\alpha_{0}+\alpha_{1}{\mathrm{DIG}}_{\mathrm{it}}+\alpha_{2}X_{\mathrm{it}}+u_{t}+v_{i}+\varepsilon_{\mathrm{it}}$$

In Equation 1, i denotes the region, t signifies the year, and α_0_ is a constant term. The development levels of digital villages and the rural older adult care service industry are denoted by DIG and IND, respectively. X_it_ represents other control variables, while u stands for the time fixed effect and v for the individual fixed effect (FE). The term ε_it_ refers to the random error term.

#### 3.1.2. Threshold effect model

As the development of the rural older adult care service industry is affected by digitalization under the influence of rural human capital, a stage-like characteristic may exist, implying a potential nonlinear relationship between these variables. To investigate the existence of this nonlinear relationship, which would validate Hypothesis 2, this study applies a panel threshold regression model (54). Consequently, we construct the following panel threshold model using rural human capital as the threshold variable.


(2) $$\begin{array}{l} {\mathrm{IND}}_{\mathrm{it}}=\beta_{0}+\beta_{1}{\mathrm{DIG}}_{\mathrm{it}}\cdot I\left(q_{\mathrm{it}}\leq \gamma \right)+\beta_{2}{\mathrm{DIG}}_{\mathrm{it}}\cdot I\left(q_{\mathrm{it}}>\gamma \right)\\ +\beta_{3}X_{\mathrm{it}}+\varepsilon_{\mathrm{it}} \end{array}$$

(3) $$\begin{array}{l} {\mathrm{IND}}_{\mathrm{it}}=\vartheta_{0}+\vartheta_{1}{\mathrm{DIG}}_{\mathrm{it}}\cdot I\left(q_{\mathrm{it}}\leq \gamma_{1}\right)+\vartheta_{2}{\mathrm{DIG}}_{\mathrm{it}}\cdot I\left(\gamma_{1}<q_{\mathrm{it}}\leq \gamma_{2}\right)\\ +\vartheta_{3}{\mathrm{DIG}}_{\mathrm{it}}\cdot I\left(q_{\mathrm{it}}>\gamma_{2}\right)+\vartheta_{4}X_{\mathrm{it}}+\varepsilon_{\mathrm{it}} \end{array}$$

Equation (2) represents a single-threshold model, wherein the sample interval is bifurcated into two segments with respective coefficient estimates β_1_ and β_2_. On the other hand, Equation (3) characterizes a dual-threshold model, whereby the sample interval is trifurcated into three segments with associated coefficient estimates ϑ_1_, ϑ_2_, and ϑ_3_. In these models, q _it_ signifies the threshold variable of rural human capital, $I\left(\cdot \right)$ represents the indicator function, and γ, γ_1_, and γ_2_ are the estimations for the thresholds, adhering to the condition γ_1_ < γ_2_.

### 3.2. Variable selection

#### 3.2.1. Explained variables

Rural older adult care service industry (IND). In the current research, the concept of the “rural older adult care service industry” remains somewhat indistinct, largely due to the lack of clear demarcation between the “older adult care service industry” and the “older adult care business.” For the purposes of this paper, our study encompasses both these aspects under the umbrella of the rural older adult care service industry. Specifically, this pertains to a novel industrial cluster that is steered by both market forces and governmental initiatives, with the aim of fulfilling the lifestyle requirements of the older adult demographic. This includes sectors such as older adult care and nursing, health services, as well as older adult education and cultural activities.

Leveraging the theoretical framework outlined previously, in addition to insights gleaned from relevant literature (55, 56), we have constructed a robust evaluation index system to assess the development trajectory of the rural older adult care service industry (Table 1). The entropy weight method has been used to determine the weights of each index. Subsequently, we obtain a consolidated sum of the developmental levels of the rural older adult care service industry for each province, autonomous region, and municipality directly governed by the central government (collectively referred to as “provinces”) spanning the period from 2011 to 2020. The methodology for this process comprises the following steps.

**Table 1:** Rural older adult care service industry development level evaluation index system.

Variables	Sub level variables	Weight
Rural older adult care service industrial infrastructure	Number of beds in rural older adult care service institutions at the end of the year (EA)	0.0388
	Number of units of rural older adult care service institutions (EA)	0.0340
	Building area of rural older adult care service institutions (m^2^)	0.0355
	Number of center rooms of older adult activity stations (EA)	0.0394
	Number of older adult schools (EA)	0.0611
Rural older adult care service industrial service output	Number of associations for older adult individuals (EA)	0.0621
	Number of aging business units (EA)	0.0260
	Number of year-end employees of rural older adult care service institutions (EA)	0.0306
	Number of employees of rural older adult care service institutions with a bachelor’s degree or above (EA)	0.0404
	Cumulative number of qualified appraisers (EA)	0.0793
	Number of adopters in rural older adult care service institutions at the end of the year (EA)	0.0473
Rural older adult care service industrial operating capital	Original value of fixed assets of rural older adult care service institutions (10000 yuan)	0.0387
	Total current year income of rural older adult care service providers (10000 yuan)	0.1111
	Total current year expenditures of rural older adult care service institutions (10000 yuan)	0.0953
Rural older adult care service industry basic security	Rural minimum living security expenditure (10000 yuan)	0.0216
	Number of older adult citizens receiving the older adult citizen allowance (EA)	0.0467
	Overall expenditure of budgeted funds for the welfare of older adult individuals (10000 yuan)	0.0737
	Aging affairs (10000 yuan)	0.0470
	Aging affairs agency subsidy level (yuan/unit/year)	0.0714

In the first step, the original indexes are standardized:


$$X_{\mathrm{ijt}}=\frac{X_{\mathrm{ijt}}-\min\left(X_{\mathrm{jt}}\right)}{\max\left(x_{\mathrm{jt}}\right)-\min\left(x_{\mathrm{jt}}\right)}\times 10\;\left(+\right)\;$$

(4) $$X_{\mathrm{ijt}}=\frac{\max\left(X_{\mathrm{jt}}\right)-X_{\mathrm{ijt}}}{\max\left(X_{\mathrm{jt}}\right)-\min\left(X_{\mathrm{jt}}\right)}\times 10\;\left(-\right)$$

Equation (4) is representative of the normalization of the indicators where t denotes the year, *i* represents the province (*i* = 1, 2, …, m; m stands for the number of provinces), and *j* symbolizes the initial indicator (*j* = 1, 2, .., n; n is the count of indicators) for a given year t after normalization. In this equation, $\;x_{ijt}$ represents the raw indicator, $\max\left(x_{jt}\right)$ is the maximum value of the first indicator across all years, and $\min\left(x_{jt}\right)$ signifies the minimum value. Given that all the indicators within the system are positively oriented, we employ the formula for the standardization of positive indicators.

In the second step, the weight of the jth indicator of the province in year t $\;S_{ijt}$ is calculated:


(5) $$S_{\mathrm{ijt}}=X_{\mathrm{ijt}}/\overset{m}{\underset{i=1}{\sum }}X_{\mathrm{ijt}}$$

In the third step, the entropy value of the first j entropy value of the index $\;E_{jt}$ is calculated, where q is the product of the span of years and the number of provinces, i.e., 300.


(6) $$E_{\mathrm{jt}}=-1/\ln q\;\overset{m}{\underset{i=1}{\sum }}P_{\mathrm{ijt}}\ln P_{\mathrm{ijt}}$$

In the fourth step, the weight of the first indicator in year t j is calculated.


(7) $$W_{\mathrm{jt}}=Y_{\mathrm{jt}}/\overset{n}{\underset{j=1}{\sum }}Y_{\mathrm{jt}},Y_{\mathrm{jt}}=1-E_{\mathrm{jt}}$$

The fifth step is to calculate the comprehensive index of the development level of the rural older adult care service industry $\;{\mathrm{IND}}_{\mathrm{it}}$ , the value of which ranges from 0 to 10. The larger the value is, the higher the level of rural older adult care service industry will develop, and vice versan


(8) $${\mathrm{IND}}_{\mathrm{it}}=\overset{n}{\underset{j=1}{\sum }}W_{\mathrm{jt}}\times X_{\mathrm{ijt}}$$

The results of calculating the weights of each index of the rural older adult care service industry system are shown in Table 1.

#### 3.2.2. Core explanatory variables

Digital Rural Development (DIG). The current evaluation index system for digital villages remains underdeveloped. As such, this study synthesizes insights and approaches from several researchers (10, 17, 57) to construct a comprehensive evaluation index system for digital villages (Table 2). Using the entropy weight method, we calculate the digitalization index for rural areas in each province from 2011 to 2020. This index serves as a measure of rural digitalization level (the specific calculation method aligns with that used for determining the development of the rural older adult care service industry).

**Table 2:** Comprehensive evaluation index system and weights of the rural digitalization level.

Variables	Sub level variables	Weight
Rural digital infrastructure	Rural mobile phone ownership (EA, +)	0.0202
	Rural home computer ownership (EA, +)	0.0437
	Rural Internet penetration rate (%, −)	0.0700
	Agricultural weather observation stations (EA, +)	0.0347
Digitalization of rural industries	Share of administrative villages with Internet broadband service (%, +)	0.0063
	Online retail sales of agricultural products (billion yuan, +)	0.2883
	Rural digital financial inclusion index (+)	0.0352
Rural digital industrialization	Average population served by rural postal business outlets (10,000 people, −)	0.0107
	The number of Taobao villages (EA, +)	0.4909

#### 3.2.3. Threshold variables

Rural Human Capital (HUM). This variable is primarily quantified using years of education and income. The years-of-education approach estimates the stock level of human capital *via* the average years of education per person in rural areas (HUM1). The income method represents actual human capital in rural areas, discounted by the cost of living index, and is split into *per capita* human capital in rural labor (HUM2) and *per capita* human capital in rural areas (HUM3). These measures are examined comparatively.

#### 3.2.4. Control variables

1. Living Standards of Rural Residents (LIV). This variable is represented by the electricity consumption in rural areas of different provinces (logged). The development of the rural older adult care service industry is somewhat contingent on the living standards of residents, with older adultly individuals who enjoy a higher quality of life being more likely to opt for old-age care (58). Rural electricity consumption refers to the cumulative annual electricity usage by rural residents for both production and lifestyle needs. This indicator, by indirectly demonstrating the accessibility of electricity in rural areas as well as the adoption and usage rates of household appliances in rural homes, offers a comprehensive reflection of the living standards of residents in rural areas.
2. Level of agricultural development (AGR). Measured by the ratio of agricultural added value to regional GDP in each province, this soft indicator has a profound impact on the rural older adult care service industry. At the same time, promoting the integration of the modern service industry and agriculture is an effective way to improve the development of the rural older adult care service industry (59).
3. Local government attention to undertakings on aging (GOV). Using the local government work report of each province over the 2011–2020 time span, the frequency of 30 keywords related to old-age care is measured (taking the logarithm) by referring to government texts and literature-related keyword research (60). Although local government policies play an important role in improving the capacity of older adult care services, the role of excessive government attention in improving the quality of older adult care services is not obvious (61).
4. Rural logistics level (LOG). Measured by the length of rural delivery lines in each province (take logarithm), to a certain extent, this variable can reflect the integrated construction of urban and rural passenger and freight transport infrastructure (62), that is, the logistics level. Additionally, the logistics level can affect the development of the tertiary industry and drive the rural older adult care service industry to effectively fit local economic development (63).
5. Ecological environment level (ENV). Measured by the forest coverage in each province, this variable can reflect the abundance and greening of regional forest resources. The ecological environment has a potential impact on industrial development (64).

### 3.3. Data resources

This study utilizes panel data from 2011 to 2020, encompassing 30 provinces in China, for analysis. Due to considerations regarding data availability and comparability, Tibet, Hong Kong, Macao, and Taiwan have been excluded for the time being. The primary sources of data include the China Statistical Yearbook, the China Rural Statistics Yearbook, the China Civil Affairs Statistics Yearbook, the China Environment Statistics Yearbook, provincial statistical yearbooks, and reports from local provincial governments. Notably, the “Rural Digital Inclusive Finance Index,” part of the comprehensive rural digital level evaluation index system, is derived from the Peking University Digital Inclusive Financial Index (2011–2020). Data pertaining to rural human capital is obtained from the China Human Capital Report 2021, released by the China Human Capital and Labor Economic Research Center of the Central University of Finance and Economics. This report provides a scientifically rigorous and comprehensive source for measuring urban and rural human capital across China and its provinces. Additional baseline data, such as the “Agriculture, Rural Areas and Farmers Database,” “Civil Affairs Database,” “Macroeconomic Database,” and “China Urban and Rural Construction Database,” were primarily retrieved from the EPS data platform. The descriptive statistical results of the pertinent variables are displayed in Table 3.

**Table 3:** Descriptive statistical results of the relevant variables.

Variables	Number of samples	Mean	Std. Dev.	Min	Max
The development level of the rural older adult care service industry (IND)	300	1.195	0.825	0.094	5.292
Rural digital index (DIG)	300	1.208	1.059	0.228	8.392
Years of education *per capita* in rural areas (HUM1)	300	7.843	0.626	5.923	10.160
Rural labor *per capita* human capital (HUM2)	300	14.641	5.607	5.934	38.384
Rural *per capita* human capital (HUM3)	300	20.922	11.776	8.149	80.497
Living standards of rural residents (LIV)	300	4.869	1.322	1.404	7.606
Level of agricultural development (AGR)	300	0.099	0.053	0.003	0.258
Local government attention to undertakings on aging (GOV)	300	2.164	0.431	0.000	3.135
Rural logistics level (LOG)	300	11.447	0.897	8.515	12.658
Ecological environment level (ENV)	300	34.573	18.070	4.000	67.000

## 4. Analysis of empirical results

### 4.1. Benchmark regression analysis

Reliability testing is crucial in this study because it helps identify potential issues with the data or methods that could introduce biases, reduce the accuracy of estimates, or affect the interpretation of the results. To assess the reliability of our data and models, we employ three methods: the variance inflation factor (VIF) to detect multicollinearity among explanatory variables, the Lagrange multiplier (LM) test to examine omitted variable bias, and the Hausman test to determine the suitability of a fixed effects model or a random effects model for our panel data. These tests provide a comprehensive approach to ensuring the robustness and validity of the findings.

The VI*F* values in Table 4 indicate that the largest value is 3.40, which is well below the threshold of 10. Thus, there is no significant concern regarding multicollinearity among variables. To ensure more precise estimation outcomes, several regression methodologies, including mixed regression, Fixed Effects (FE) model, and Random Effects (RE) model, are employed to scrutinize the quantitative associations between the variables. The selection of these three models is guided by the Lagrange Multiplier (LM) test and the Hausman test. The results of the regression analyzes are presented in Table 5. The test results confirm that all the above-mentioned tests are statistically significant at the 1% level, suggesting the Fixed Effects (FE) model is the optimal choice for this study. To mitigate the influence of unobservable individual and temporal effects on the model, a two-way FE model has been chosen.

**Table 4:** VIF of each interpretation variable.

	DIG	LIV	AGR	GOV	LOG	ENV	Mean value
VIF	1.460	3.400	1.920	1.200	2.600	1.190	1.960
1/VIF	0.683	0.294	0.520	0.832	0.384	0.837	0.592

**Table 5:** Benchmark regression results.

Variables	OLS model	RE model	FE model
DIG	0.079**	0.044*	0.059**
	(2.15)	(1.88)	(2.07)
LIV	0.206***	0.326***	0.360***
	(4.60)	(4.86)	(3.72)
AGR	−2.088**	2.394*	3.993**
	(−2.50)	(1.75)	(2.00)
GOV	−0.187**	−0.101**	−0.048
	(−2.29)	(−2.16)	(−0.99)
LOG	0.393***	0.251***	0.260***
	(6.81)	(3.32)	(3.01)
ENV	−0.002	−0.004	0.044**
	(−1.01)	(−0.76)	(2.37)
Constant	−3.731***	−3.202***	−5.401***
	(−7.11)	(−4.12)	(−4.48)
Province	No	No	Yes
Year	No	No	Yes
R-squared OBS	0.555300	0.502300	0.901300
LM test		656.29***	
Hausman test			36.75***

The corresponding t value is in parentheses; ***, ** and * indicate significance at the 1, 5 and 10% levels, respectively. The same applies below.

Estimation results pertaining to digital villages reveal a positive and statistically significant impact coefficient on the development of the rural older adult care service industry at the 5% level. This supports the proposition that digital villages foster the development of this sector, which indicates that the construction of digital villages can empower the rural older adult service industry in four dimensions: infrastructure, service output, operating capital, and basic security. This finding is consistent with the previous theoretical analysis (32, 37, 42, 45), thereby validating Hypothesis 1. An analysis of the control variable estimation results, barring local government attention to aging, shows estimation coefficients to be positive and statistically significant at least at the 5% level. These findings suggest these variables favor the development of the rural older adult care service industry. The detailed results of the benchmark regression is presented in Table 5.

### 4.2. Panel threshold effect test

This study estimates whether there is a single threshold effect, a double threshold effect or a triple threshold effect for years of education per rural person, human capital per rural person in the labor force and human capital per rural person in each of the 30 provinces. Drawing from Hansen’s “bootstrap” approach, Stata17.0 statistical software is utilized. Through 1,000 sample iterations, the corresponding *p*-value for the test statistics is ascertained to determine the presence of a threshold effect. The test outcomes are presented in Table 6.

**Table 6:** Results of the threshold effect test.

Threshold variables	Number of thresholds	F value	*p* value	The critical value		
				10%	5%	1%
HUM1	Single threshold (ST)	28.58**	0.0270	17.7468	23.3798	38.0588
	Double threshold (DT)	6.16	0.6120	18.6410	25.9857	44.9800
	Triple threshold (TT)	9.92	0.5020	24.3354	29.3992	42.9645
	Single threshold (ST)	37.23**	0.0160	21.3221	28.0817	43.4262
HUM2	Double threshold (DT)	38.27***	0.0040	16.9331	19.4378	29.3987
	Triple threshold (TT)	9.73	0.3550	30.0915	45.0284	80.4525
	Single threshold (ST)	40.15**	0.0150	22.7366	27.6656	44.0092
HUM3	Double threshold (DT)	36.73***	0.0050	18.9531	22.9369	32.9674
	Triple threshold (TT)	21.33	0.1320	27.9115	47.9032	82.0057

The *P* value is the probability value obtained by repeatedly sampling 1,000 times using the bootstrap method, and the value is used to judge how significant the F statistic is and whether the threshold effect test is passed. The following table is the same.

As indicated in the preceding table, the rural *per capita* years of education passes the single threshold effect test, suggesting the need to construct a panel single threshold measurement model. Both the rural *per capita* labor force human capital and the rural *per capita* human capital clear the double threshold effect test, implying the necessity to establish a panel double threshold measurement model. Specific threshold estimation results are presented in Table 7.

**Table 7:** Estimation results of the threshold value.

Threshold variables	Type of threshold	Threshold value	95% confidence interval
HUM1	The first threshold	8.4402	(8.3762, 8.4457)
HUM2	The first threshold	17.5195	(17.2507, 17.5693)
	The second threshold	24.9890	(24.2645, 29.8457)
HUM3	The first threshold	22.4873	(22.3392, 22.6134)
	The second threshold	38.1347	(37.5252, 64.4969)

Based on the threshold value estimation, the single estimated threshold value stands at 8.4402 when the *per capita* years of education in rural areas is utilized as the threshold variable. Using the human capital of the rural *per capita* labor force as the threshold variable yields double estimated threshold values of 17.5195 and 24.9890. Similarly, applying the *per capita* human capital in rural areas as the threshold variable results in double estimated threshold values of 22.4873 and 38.1347, which are significant at the 5% level. To verify the validity of the threshold estimation, the likelihood ratio (LR) is considered. Following the principle of the threshold model, the threshold estimation corresponds to the value where the LR approaches 0. Consequently, the LR function graph corresponding to the threshold estimation within the 95% confidence interval can be derived.

**Figure 1:**
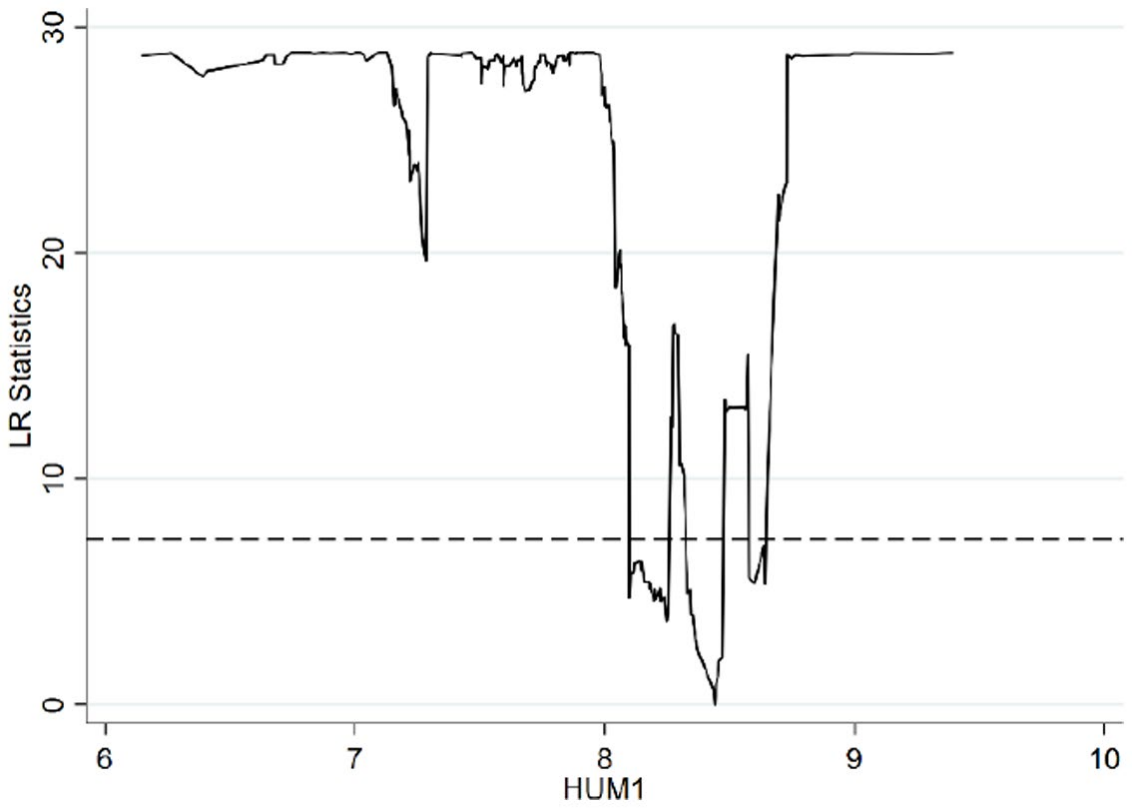
Estimation results when HUM1 is a single threshold variable.

**Figure 2:**
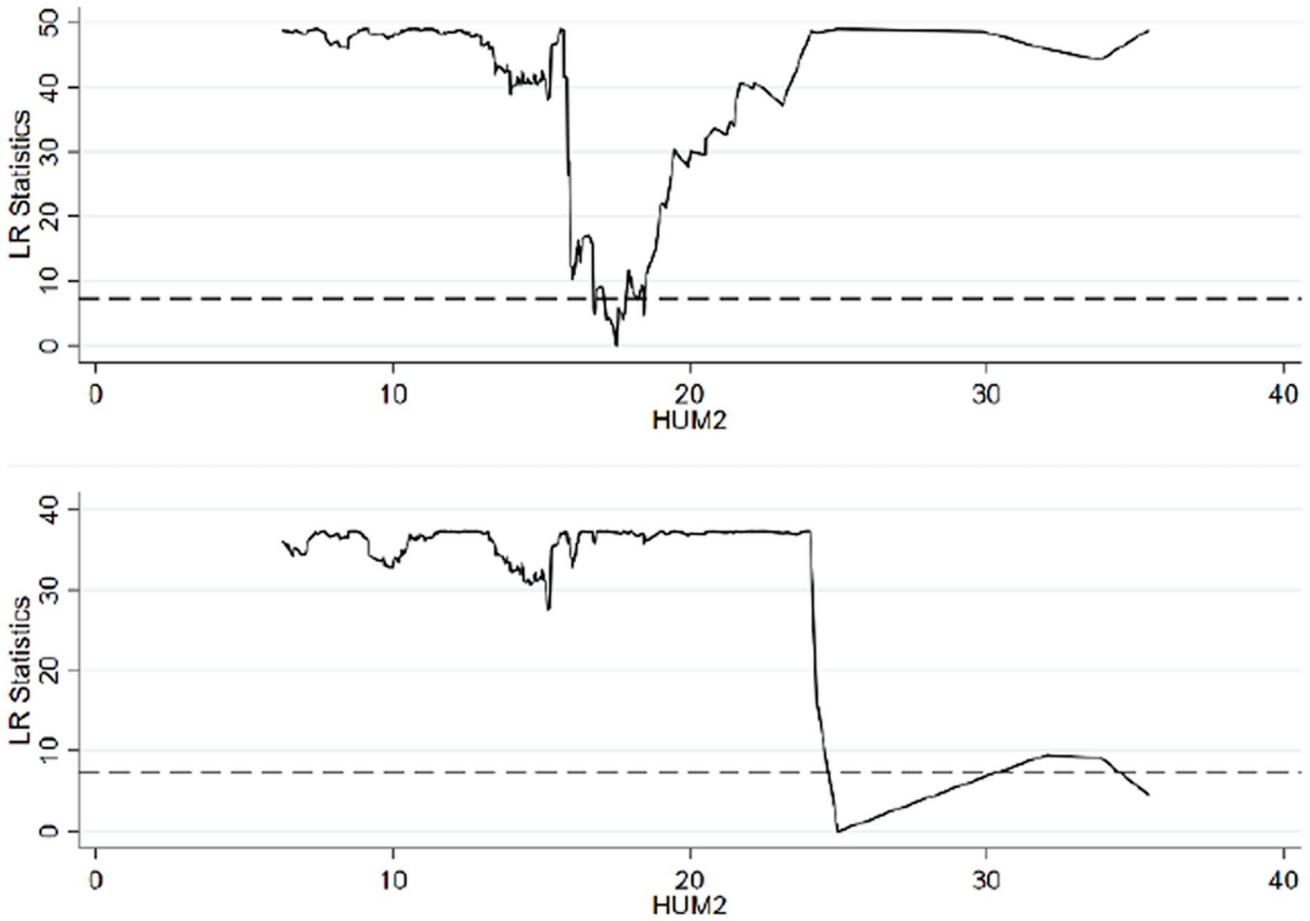
Estimation results when HUM2 is a double threshold variable.

**Figure 3:**
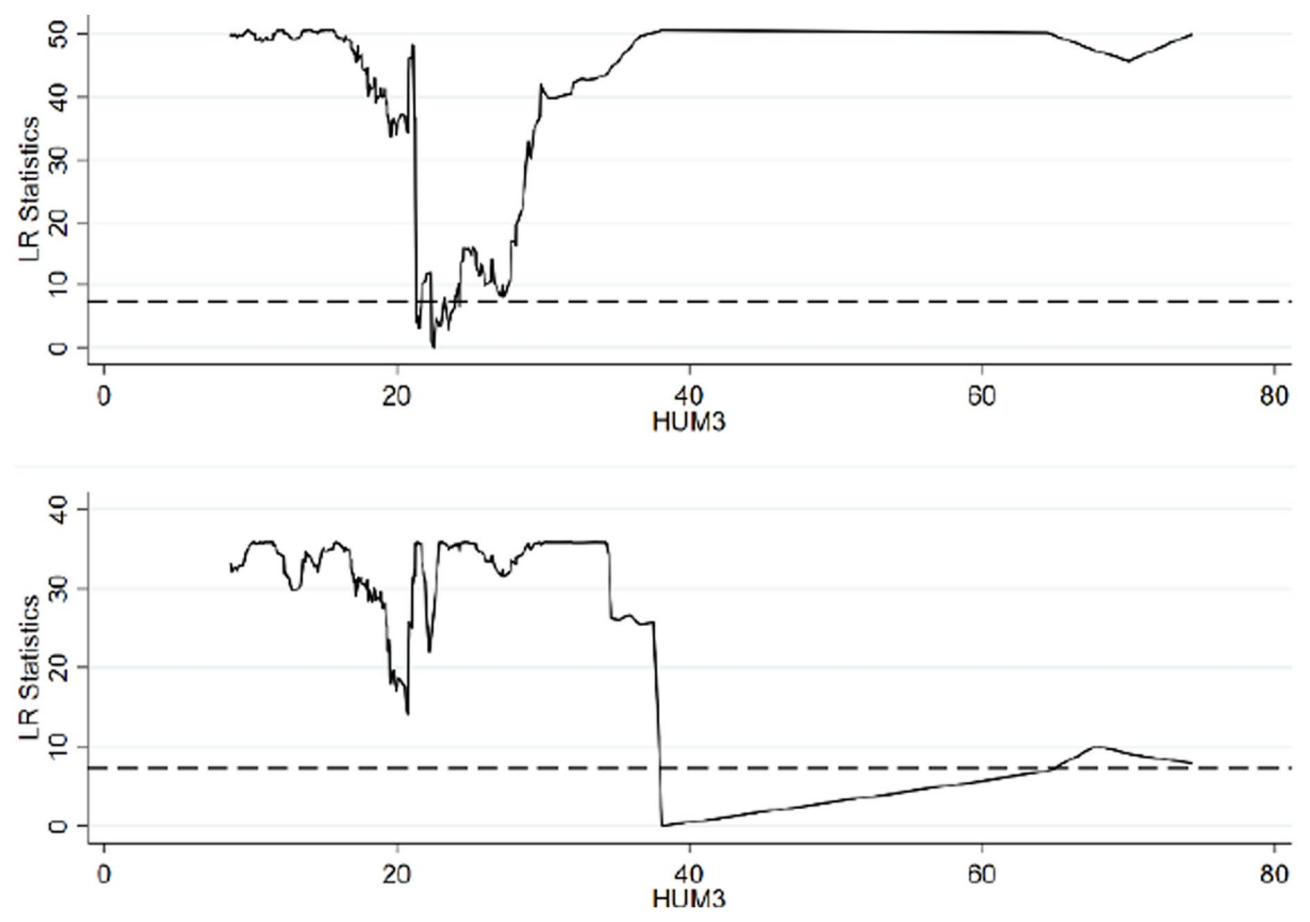
Estimation results when HUM3 is a double threshold variable.

In alignment with Table 7 and Figure 1 illustrates the LR function of the threshold estimate for rural *per capita* years of education, which stands at 8.4402, within the 95% confidence interval. Figure 2 portrays the LR function of the estimated threshold values for rural *per capita* labor force human capital, positioned at 17.5195 and 24.9890, also within the 95% confidence interval. Figure 3 delineates the LR function of the estimated threshold values for rural *per capita* human capital, at 22.4873 and 38.1347. Within these, the lowest point of the LR statistics signifies the corresponding actual threshold, while the dotted line denotes a critical value of 7.3523. Given that the critical value substantially exceeds the threshold value, the aforementioned threshold value is verified as genuine and valid.

During the process of threshold effect testing, the parameter estimation results of the panel threshold model are also derived, as presented in Table 8. Based on these estimates, when the *per capita* years of education in rural areas serves as the threshold variable, it only achieves significance at the 1% level after surpassing the threshold of 8.4402. This result emphatically underscores the necessity of promoting nine-year compulsory education in rural regions of China. Furthermore, it is only through the continuous enhancement of both the scope and intensity of rural education that digital technology can be effectively integrated into rural older adult care services.

**Table 8:** Estimation results of threshold model parameters.

Variables	Model (1)	Variables	Model (2)	Variables	Model (3)
Threshold variables	HUM1	Threshold variables	HUM2	Threshold variables	HUM3
LIV	0.327***	LIV	0.213**	LIV	0.160*
	(3.64)		(2.40)		(1.80)
AGR	4.413**	AGR	3.528**	AGR	1.234
	(2.58)		(2.15)		(0.75)
GOV	−0.088*	GOV	−0.079*	GOV	−0.108**
	(−1.94)		(−1.81)		(−2.57)
LOG	0.241***	LOG	0.229***	LOG	0.189**
	(2.91)		(2.89)		(2.44)
ENV	−0.007	ENV	−0.021	ENV	−0.020
	(−0.49)		(−1.55)		(−1.52)
HUM1(q_it_ ≤ γ)	−0.005	HUM2(q_it_ ≤ γ_1_)	0.224***	HUM3(q_it_ ≤ γ_1_)	0.234***
	(−0.19)		(6.38)		(6.79)
HUM1(q_it_ > γ)	0.141***	HUM2(γ_1_ < q_it_ ≤ γ_2_)	−0.001	HUM3(γ_1_ < q_it_ ≤ γ_2_)	−0.011
	(4.88)		(−0.04)		(−0.49)
		HUM2(q_it_ > γ_2_)	0.502***	HUM3(q_it_ > γ_2_)	1.056***
			(4.29)		(5.70)
Constant	−3.198***	Constant	−2.131**	Constant	−1.158
	(−3.14)		(−2.15)		(−1.17)

When the human capital of the rural *per capita* labor force is utilized as the threshold variable, the regression coefficient of rural digitalization stands at 0.224 and is significant at the 1% level when the human capital of the rural *per capita* labor force is below the first threshold of 17.5195. As rural *per capita* labor force human capital exceeds the first threshold and remains between the first and the second thresholds, the regression coefficient of rural digitalization transforms to-0.001 and loses significance. However, as the rural *per capita* labor force human capital surpasses the second threshold of 24.9890, the regression coefficient of rural digitalization not only retains its positive significance at the 1% level but also further escalates to 0.502.This evidence emphatically demonstrates that an elevated level of human capital in the rural *per capita* labor force strengthens its capacity to leverage digital resources and technology, thus enhancing the efficiency of digital empowerment in rural older adult care service industries. Viewing the regression results of the control variables, there is minimal deviation from the baseline regression results (Table 5) after accounting for the threshold effect, thereby substantiating that these factors facilitate the development of the rural older adult care service industry. The estimation process with rural *per capita* human capital as the threshold variable parallels the aforementioned procedure and will not be further dissected here.

The threshold effect test elucidates a conspicuous nonlinear threshold relationship between digital villages, human capital, and the growth of the rural older adult care service industry. Specifically, the efficacy of digital technology in catalyzing the development of the rural older adult care service industry is contingent upon and influenced by the level of rural human capital. A higher level of human capital in rural areas optimizes the role of rural digitalization in fostering integrated rural industrial development. As discussed in the previous section of the theoretical analysis, the efficacy of new technologies in empowering traditional industries is limited by human capital, and this holds true for the rural older adult care service industry as well. The degree of acceptance of digital technology by service providers will undoubtedly impact the empowerment of the digital village, and this influence mechanism will also exhibit regional variations, which aligns with the findings of the previous section (46, 51–53). Therefore, corroborating the previously established research Hypothesis 2. Concurrently, whether the metric is years of education or rural human capital as gaged by income, the alteration trend of the rural digital estimation coefficient remains fundamentally consistent, which bolsters the robustness of the aforementioned conclusion to a certain extent.

### 4.3. Coupling coordination test

The enhancement of the digital rural older adult care service industry is bounded by the level of human capital, with increased rural digitalization typically magnifying the spillover effect of human capital on the industry’s development. Digital villages and the rural older adult care service industry reciprocally influence each other, exhibiting a dynamic coupling relationship. Building upon this, we employ the coupling coordination model in this paper to further validate the interplay between digital villages and the rural older adult care service industry, and the factors influencing them. Guided by the findings from related research (65), this paper present the coupling coordination model of the two systems as follows:


(9) $$D=\sqrt{C*T}$$

In the formula,


(10) $$C={\left\{\left(U_{1}\times U_{2}\right)/{\left(\left(U_{1}+U_{2}\right)/2\right)}^{2}\right\}}^{r}$$

In Equation (10), $U_{1}\;$ represents digital villages; $U_{2}$ represents the rural older adult care service industry; and r is the mediation coefficient, which in this article is set to 2.


(11) $$T=\alpha U_{1}+\beta U_{2}$$

In Equation (9), D represents the correlation degree; its value is (0,1). C is the coupling degree, and T is the coordination degree. In the coupling coordination model of the two systems, it is generally assumed that the two subsystems are equally important. Thus, the values of α and β in Equation (11) are 0.5.

Using the above methods, it is found that, from a nationwide perspective, the coupling coordination degree (D) of China’s digital villages and the rural older adult care service industry during the sample period amounts to 0.2950, indicating a low level of coupling coordination (66). Regionally, the eastern area exhibits the highest degree of coupling (D = 0.3348), followed by the central region (*D* = 0.3137). The northeast (*D* = 0.2623) and western regions (*D* = 0.2569) display the lowest degrees and fall below the national average, suggesting that regional digitalization contributes to the heterogeneity of the rural older adult care service industry. Following this, the three-dimensional rural human capital and the coupling coordination degree values are analyzed, and the results are shown in Table 9. The *per capita* years of education, the *per capita* labor force human capital, and the *per capita* human capital in rural areas all show significantly positive correlations at the 1% level. This evidence suggests that a high level of human capital fosters the coupling of digital villages and the rural older adult care service industry. Moreover, the enhancement role of human capital for the *per capita* education level is more pronounced, further validating the reliability of human capital structure heterogeneity in the prior region (11, 13, 25).

**Table 9:** Coupling coordination regression results.

Variables	Model (1)	Model (2)	Model (3)
HUM1	0.070***		
	(5.53)		
HUM2		0.011***	
		(3.72)	
HUM3			0.009***
			(5.12)
LIV	0.049***	−0.011	−0.023*
	(3.59)	(−0.87)	(−1.71)
AGR	−1.653***	−0.591**	−0.372
	(−6.54)	(−2.33)	(−1.46)
GOV	−0.003	−0.009	−0.010*
	(−0.41)	(−1.43)	(−1.70)
LOG	0.023*	0.028**	0.029***
	(1.82)	(2.51)	(2.72)
ENV	0.004*	−0.004	−0.004*
	(1.81)	(−1.65)	(−1.65)
Constant	−0.728*** (−4.68)	0.083 (0.52)	0.060 (0.39)
Province	Yes	Yes	Yes
Year	Yes	Yes	Yes
OBS	300	300	300
*R*-squared	0.833	0.886	0.891

## 5. Discussion

In summary, this study’s baseline regression findings demonstrate that the development of digital villages significantly benefits the progression of the rural older adult care service industry. Over recent years, the degree of rural digitalization and the developmental stage of rural older adult care services across China have witnessed considerable enhancement, with the continuous improvement in rural digitalization serving as a crucial catalyst for the ongoing intelligent evolution of the rural older adult care service industry (5). Elevated living standards of rural residents stimulate demand for high-quality older adult care services, a demand that is met with the requisite supply of services (44). Furthermore, a higher degree of agricultural development, logistics, and ecological environment fosters the evolution of the rural older adult care service industry (58). Specifically, superior agricultural development levels supply ample production factors to the rural older adult care service industry, spurring integration between industries (59). Improvements in rural logistics ensure the reach of the older adult care service industry into rural areas, while a high-quality ecological environment provides an optimal backdrop for expanding the rural older adult care service industry chain (62–64). These results are consistent with existing research. Notably, local governments demonstrate no significant attention toward aging-related matters. This suggests that while the government’s “visible hand” can supply resource elements essential for industry development, the expansion of governmental boundaries may also encroach upon the operational space of the market economy. This could result in resource misalignment within the older adult care service industry, impeding the industry’s operational efficiency from reaching the Pareto improvement path. Given the real-world context of rural areas in China, the current level of standardization within the rural older adult care service industry remains inadequate, and some government intervention measures struggle to fulfill their intended roles.

The observed threshold effect results raise a pertinent question warranting detailed exploration. While digital villages bear substantial potential for the rural senior care service industry, actualizing this potential necessitates a particular degree of technical competency (25). In essence, the facilitative influence of digital technology on the development of the rural senior care service industry is contingent upon and impacted by the level of rural human capital. The more advanced the level of rural human capital, the more effective the construction of digital villages becomes in advancing the development of rural industrial integration. The result is consistent with the research results of human capital (11, 15, 50). Given the population outflow in rural areas of China, specifically the youth’s migration to urban centers, we confront a growing issue of aging and a dearth of technological literacy. This situation inevitably impacts our overall recommendations and creates hindrances in accomplishing our anticipated objectives (1). One feasible strategy to tackle this issue entails offering specialized technological training programs for the older adults, ensuring they are equipped with adequate knowledge and skills to navigate these systems (27). We could also employ the young generation as conduits for technology transfer, leveraging the aid of their offspring, kin, or community members to facilitate the older adults in understanding and utilizing these technologies (29). While promoting high-tech agriculture aids infrastructure development and heightens economic productivity, additional research and practical investigations are indispensable to ascertain these technologies’ genuine impact on the older adults (12). Consequently, forthcoming research needs to acknowledge and address these obstacles to fully tap into the potential of digital village construction for the rural older adult care service industry.

The findings of the coupling coordination test indicate that, broadly speaking, the linkage between digital village development and the rural senior care service industry is considerably weak across China’s rural regions (17). However, augmenting human capital can stimulate a better integration between these two systems. In fact, current developments suggest that as digital technology permeates deeper into rural areas, it will invariably lead to a differentiated impact due to the digital divide (67). Therefore, bridging the regional human capital gap becomes key to developing digital intelligence within the rural older adult care service industry. The initial step in bridging the regional human capital gap is to enhance the construction of digital infrastructure in rural areas based on local needs, establishing digital transmission channels for the older adult care industry and expanding the coverage of the digital older adult care service platform (22). These measures aim to provide personalized care for more older adults and broaden the scope of industrial development. On this basis, the government should hasten the training of new digitally-driven professional farmers, enhance data technology training for the rural labor force, improve their ability to apply digital resources and technology to fortify the link between digital villages and the rural older adult care service industry, promoting the in-depth development of the industry (43).

From a theoretical standpoint, this study first uncovers the existence of a human capital threshold effect during the process of digital village empowerment of the rural older adult care service industry (31). This extends the application of human capital theory and offers a fresh theoretical perspective for comprehending and explaining the emerging dynamics of the rural older adult care service industry’s development. Secondly, the study further refines the mechanics of the human capital threshold effect, including the influence of factors such as the living standards of rural residents, the level of agricultural development, logistics facilities, and the ecological environment (55). This insight helps deepen our understanding of the specific role and extent of the human capital threshold effect’s influence within the rural older adult care service industry. Lastly, by comparing the empowerment effects under varying levels of human capital, the study exposes the critical role of human capital levels in the empowerment process, thereby substantiating the adaptability and flexibility of human capital theory in practical application.

From a practical viewpoint, our research findings provide essential perspectives for advancing the sustainable progression of the rural older adult care service industry (18). Firstly, it is vital to place high emphasis on bolstering infrastructure pertinent to production and lifestyle, such as agriculture, water conservation, transportation, and telecommunications. Such enhancements will expedite the creation of digital villages, thereby mobilizing the productive capabilities of digital components (44). The digital metamorphosis of the rural older adult care service industry should be primarily market-driven. Secondly, to fully capitalize on the role of digital villages in advancing the rural older adult care service industry, considerable focus should be directed toward the state of human capital and its regional disparities. Different areas should consider their local circumstances in relation to rural education development. Resource allocation, inclusive of human and financial investments, should target rural areas with an emphasis on the critical role of education in accumulating human capital and escalating the level of human capital in rural areas. Finally, underlining the integration and growth of digital villages with the rural older adult care service industry is paramount. Rural areas with poorly coupled two systems, priority should be shifted toward developing fundamental older adult care services. This approach is preferable to a hasty pursuit of digital transformation, which could inadvertently place further strain on the regional economy.

There are also some shortcomings in this study, including the use of a restricted indicators to appraise the variables. Limited to data availability, the indicator system was designed mainly incorporating components from pertinent studies, which may result in not covering all aspects thoroughly. Thus, future research should strive to create a more balanced indicator system. Another challenge arises from the vast range of fields encompassed by the digital village and rural older adult care service industry. Consequently, our findings may lack detailed and specific reference points, a common occurrence in empirical research. This implies that future research should broaden the study’s focus and explore more specialized areas within these fields. The final hurdle concerns the data source for this study, rural areas across diverse provinces in China. The findings’ universal applicability may be limited as the samples may not represent the global context accurately. Thus, to enhance the universality and representativeness of the findings, future research should aim to include a wider, more diverse range of data sources.

## 6. Conclusion

In conclusion, this study reveals significant insights about the role of the digital village in the enhancement of the rural older adult service industry. The analysis reveals that digital villages can notably empower the rural older adult care service industry. Enhancements in aspects like rural living standards, agricultural development, logistics, and ecological environments are shown to significantly contribute to the development of this industry. Our study also identifies a remarkable threshold effect of human capital on the empowerment potential of digital villages on the rural older adult care service industry. This finding underlines the necessity to foster and boost human capital to fully utilize the potential of digital villages for the betterment of the rural older adult care service industry. Moreover, the research indicates a generally low coupling coordination between the digital village system and the rural older adult care service industry system. It is evident from the results that disparities in human capital levels significantly impact the coupling of the two systems. To achieve a more harmonized and effective integration, it is essential to bridge the human capital gap and enhance the coupling between the two systems. These findings not only highlight the significance of digital villages and human capital in bolstering the growth of rural older adult care service industry but also underscore the need for targeted policies and strategies to amplify their potential. This study, therefore, provides an important foundation for further research and policy development in this area.

## Data availability statement

The original contributions presented in the study are included in the article/supplementary material, further inquiries can be directed to the corresponding author.

## Author contributions

SL: conceptualization. SZ: formal analysis. ZH and SZ: methodology. CL: supervision. All authors have read and agreed to the published version of the manuscript.

## Funding

This work was supported by the National Natural Science Foundation of China (NSFC No. 71962005), and the Innovation Project of Guangxi Graduate Education (YCSW2023357).

## Conflict of interest

The authors declare that the research was conducted in the absence of any commercial or financial relationships that could be construed as a potential conflict of interest.

## Publisher’s note

All claims expressed in this article are solely those of the authors and do not necessarily represent those of their affiliated organizations, or those of the publisher, the editors and the reviewers. Any product that may be evaluated in this article, or claim that may be made by its manufacturer, is not guaranteed or endorsed by the publisher.
